# Microbe Profile: Streptomyces venezuelae – a model species to study morphology and differentiation in filamentous bacteria

**DOI:** 10.1099/mic.0.001541

**Published:** 2025-03-13

**Authors:** Max L. Jordan, Susan Schlimpert

**Affiliations:** 1 Department of Molecular Microbiology, John Innes Centre, Colney Lane, Norwich, NR4 7UH, UK; 2 Centre for Microbial Interactions, Norwich Research Park, Norwich, NR4 7UH, UK

**Keywords:** *Actinomycetota*, cell division, multicellular development, sporulation

## Abstract

*Streptomyces* bacteria are renowned for their multicellular lifestyle and production of bioactive molecules (natural products) with important applications in medicine, agriculture and industry. Studies of several *Streptomyces* species have provided a foundational understanding of their biology and metabolism. However, investigating the spatiotemporal processes governing the morphogenesis and development of these remarkable bacteria has been technically challenging due to their complex life cycle. The adoption of *Streptomyces venezuelae* as a new experimental model species has overcome these limitations and opened the door to fully explore the regulation and cell biology of *Streptomyces* development. A key advantage of *S. venezuelae* is its ability to complete its entire life cycle in liquid culture, facilitating the effective use of genome-wide analysis techniques and advanced cell biology approaches. This has provided significant new insights into the regulatory networks and molecular mechanisms underlying *Streptomyces* growth, division, developmental transitions and genome organization.

## Graphical Abstract

*Streptomyces venezuelae* undergoing the spore-to-spore life cycle in liquid culture makes it a versatile model organism for generating key insights into the regulatory networks, cell biology and development of this genus.

## Taxonomy

Phylum, *Actinomycetota* (formerly known as *Actinobacteria*); class, *Actinomycetia*; order, *Streptomycetales*; family, *Streptomycetaceae*; genus, *Streptomyces*; species, *Streptomyces venezuelae*. The species name ‘*venezuelae*’ reflects its initial isolation from a soil sample collected in Caracas, Venezuela [1].

## Properties

*S. venezuelae* is an aerobic, soil-dwelling, monoderm bacterium that was first characterized as a producer of the antibiotic chloramphenicol [2]. Subsequent work has identified the production of additional antibiotics, such as jadomycin and the pikromycins [2].

In common with other species of the genus, the *S. venezuelae* life cycle begins when spores germinate to produce vegetative hyphae. Vegetative hyphae grow by tip extension and branching, forming a dense network of interconnected filaments known as mycelium. These filaments exhibit sporadic septation that does not result in cell-cell separation, leading to the formation of interconnected compartments containing multiple decondensed chromosomes. In response to environmental and cellular signals, a reproductive growth phase is initiated, which involves a functionally distinct type of non-branching (reproductive) hyphae, also referred to as aerial hyphae. Reproductive hyphae will cease to grow and undergo an almost synchronized septation event during which these hyphae are transformed into chains of unigenomic, green-pigmented exospores that are subsequently released into the environment to restart the life cycle [2].

*S. venezuelae* is a fast-growing species that can complete its spore-to-spore life cycle in 3–4 days on a solid medium and in about 24 h in a submerged culture. As in other *Streptomyces* species, *S. venezuelae* sporulation is associated with the production of volatile compounds, notably geosmin, which attracts arthropods to aid in spore dispersal and also results in a characteristic ‘earthy’ odour [3].

## Genome

The common laboratory strain is *S. venezuelae* NRRL B-65442, which carries a GC-rich 8.2 Mb linear chromosome and 158 kb plasmid [2]. The chromosome is currently annotated to contain 7141 protein-coding sequences arranged in a pattern characteristic of *Streptomyces* chromosomes consisting of a well-conserved core region and flanking arms enriched in biosynthetic gene clusters (BGCs) required for specialized metabolism. Bioinformatic analysis of the *S. venezuelae* genome predicts the presence of 34 BGCs [2].

## Phylogeny

*S. venezuelae* NRRL B-65442 is a derivative of the type strain *S. venezuelae* ATCC 10712. A detailed history of the origin of the NRRL B-65442 isolate is documented in Gomez-Escribano *et al.* [2].

## Key features and discoveries

Unlike most *Streptomyces* species, *S. venezuelae* grows in a highly dispersed manner before sporulating abundantly and almost synchronously in liquid culture. All regulatory mutations that block sporulation on solid media also prevent sporulation in liquid culture [4]. Moreover, *S. venezuelae* is genetically amenable, with efficient systems for genetic manipulation, such as λRED or I-SceI-based mutagenesis strategies [5,6], CRISPR-Cas9-mediated genome editing [7], generalized phage transduction and bacteriophage attachment sites for the site-specific integration of donor DNA [6].

The characteristic dispersed and synchronous growth habit of *S. venezuelae* in liquid culture has enabled the effective application of global genome analysis techniques, such as ChIP-seq, RNA-seq and the mapping of chromosome contact sites (Hi-C), to characterize the regulatory networks controlling developmental transitions and chromosome organization over the entire spore-to-spore life cycle. Furthermore, the use of *S. venezuelae* facilitates the successful application of cell biology approaches, such as time-resolved fluorescence microscopy, to study central developmental processes such as growth, morphogenesis, sporulation-specific cell division and chromosome segregation at the cellular level [8].

Key discoveries underpinning *Streptomyces* development made using *S. venezuelae* as a model species include (i) the identification of the central regulatory networks that control development [8]; (ii) the importance of small signalling molecules, such as c-di-GMP in coordinating developmental processes, including hyphal differentiation and sporulation [9]; (iii) the discovery of alternative and reversible *Streptomyces* growth modes including ‘exploratory growth’ (a motility-type response, resulting in rapid colony expansion) and the formation of ‘S-cells’ (conditional shedding of the cell wall in response to hyperosmotic stress) [10,11]; (iv) the characterization of critical cellular components controlling polar growth and cell division during vegetative growth and sporulation such as ParAB, SepH and SepX [12,14]; (v) the mapping of chromosome topology during sporogenesis [15]; and (vi) the characterization of volatile organic molecules that promote exploration and spore dispersal [2,16].

## Open questions

While considerable progress has been made in understanding the complexity and molecular details that underpin *Streptomyces* biology utilizing *S. venezuelae*, many open questions remain and await further investigation to reveal how discoveries made under laboratory conditions translate to the ecology of streptomycetes. For example:

- What is the full spectrum of regulatory molecules involved in *Streptomyces* cell cycle control and intra-species interaction?
- What are the regulatory programmes that underpin environmental sensing and stress responses to ensure the ecological success of *Streptomyces* in diverse habitats?
- What novel defence mechanisms against selfish mobile genetic elements exist in the underexplored *Streptomyces*? How does the interplay of complex developmental and metabolic programmes in *Streptomyces* contribute to these unexplored immune strategies?
- How is the spatiotemporal organization of the biosynthetic machinery involved in specialized metabolite production and what are the cell biological processes that link multicellular development and antibiotic production in *Streptomyces*?

## Abbreviations

BGCs: biosynthetic gene clusters
